# Progress in Research of Chitosan Chemical Modification Technologies and Their Applications

**DOI:** 10.3390/md20080536

**Published:** 2022-08-21

**Authors:** Qizhou Chen, Yi Qi, Yuwei Jiang, Weiyan Quan, Hui Luo, Kefeng Wu, Sidong Li, Qianqian Ouyang

**Affiliations:** 1 The Public Service Platform of South China Sea for R&D Marine Biomedicine Resources, Marine Biomedical Research Institute, Guangdong Medical University, Zhanjiang 524023, China; 2 The Marine Biomedical Research Institute of Guangdong Zhanjiang, Zhanjiang 524023, China; 3 Guangdong (Zhanjiang) Provincial Laboratory of Southern Marine Science and Engineering, Zhanjiang 524023, China

**Keywords:** alkaline cationic polymer, chemical modification, chitosan, chitosan derivatives

## Abstract

Chitosan, which is derived from chitin, is the only known natural alkaline cationic polymer. Chitosan is a biological material that can significantly improve the living standard of the country. It has excellent properties such as good biodegradability, biocompatibility, and cell affinity, and has excellent biological activities such as antibacterial, antioxidant, and hemostasis. In recent years, the demand has increased significantly in many fields and has huge application potential. Due to the poor water solubility of chitosan, its wide application is limited. However, chemical modification of the chitosan matrix structure can improve its solubility and biological activity, thereby expanding its application range. The review covers the period from 1996 to 2022 and was elaborated by searching Google Scholar, PubMed, Elsevier, ACS publications, MDPI, Web of Science, Springer, and other databases. The various chemical modification methods of chitosan and its main activities and application research progress were reviewed. In general, the modification of chitosan and the application of its derivatives have had great progress, such as various reactions, optimization of conditions, new synthetic routes, and synthesis of various novel multifunctional chitosan derivatives. The chemical properties of modified chitosan are usually better than those of unmodified chitosan, so chitosan derivatives have been widely used and have more promising prospects. This paper aims to explore the latest progress in chitosan chemical modification technologies and analyze the application of chitosan and its derivatives in various fields, including pharmaceuticals and textiles, thus providing a basis for further development and utilization of chitosan.

## 1. Introduction

Chitosan, which is also known as deacetylated chitin and soluble chitin, has the chemical name (1→4)-2-amino-2-deoxy-d-glucose and belongs to a group of unbranched binary polysaccharides composed of *N*-acetyl-d-glucosamine and d-glucosamine. Chitosan is a product of the partial deacetylation of the natural polysaccharide chitin, which is mainly derived from the crustaceans of marine arthropods, such as shrimps and crabs [1,2]. Secondly, chitin exists in the exoskeleton of insect crustaceans and mollusks, the cell membranes of fungi and algae, and the cell walls of higher plants, with a storage capacity second only to cellulose. Chitosan is the only positively charged natural alkaline polysaccharide that exists in large quantities in nature [3]. In addition to good biodegradability, biocompatibility, cell affinity, blood compatibility, safety, and nontoxicity [4,5,6,7], chitosan contributes to antibacterial, hemostatic, antitumor, anti-Alzheimer’s disease, immunity, wound healing, and other biological activities [8,9,10,11,12,13,14]. Due to the huge potential of its advantageous characteristics, chitosan has long been regarded as a promising material for biomedical research applications. Widely used in textiles, beauty care, cosmetics, gene therapy, sutures, wound dressings, bioimaging, and antibacterial agents, etc. [15,16,17,18,19,20], chitosan has an especially prominent role in the preparation of sustained-release drug materials and drug carriers [21,22]. However, the large number of hydroxyl and amino active groups in the molecular structure of chitosan easily forms hydrogen bonds, which results in a compact crystal structure that is insolubility in alkaline solutions and most organic solvents. Therefore, its application is severely limited. Fortunately, −NH_2_ and −OH in the chitosan molecule are chemically active, so other groups can be introduced at these sites for structural modification to improve performance [23,24]. Modification of chitosan is a necessary process to exploit the rich biological activity of chitosan, improve its solubility, and prepare chitosan derivatives with the required physicochemical and biological properties for specific applications. At present, chitosan modification is a research hotspot of scholars. Chitosan modification methods mainly involve chemical, physical, and compound modification. New materials prepared by physical modification generally only need adjustments in physical properties and in the mechanics of raw materials. Compound modification involves preparing the composite material by combining chitosan and polymer materials, which can improve drug loading and embedding rates. In addition to improving the water solubility of chitosan, chemical modification can be compounded with antioxidant, antibacterial, antitumor, and other active substances to obtain a compound with the advantages of antitumor, antioxidation, and antibacterial properties, which in combination effectively increases the biological activity and application scope of chitosan. At present, physical modification and compound modification have few applications; mainly, chemical modification is used. For this reason, chemical modification allows for the synthesis of derivatives with controllable solubility, ionic properties, and hydrophilic and lipophilic properties, which are more suitable as biomedicine carriers [25]. Commonly used chemical modifications of chitosan include carboxylation modification, alkylation modification, esterification modification, sulfonation modification, quaternary ammonium salt modification, graft copolymerization modification, Schiff base modification, and others. This report provides a review of common chemical modification technologies used for chitosan production and the main activities and applications of chitosan and its derivatives with the aim to provide theoretical guidance for improving the physical and chemical properties of chitosan and expanding the scope of its applications.

## 2. Structural Composition of Chitosan

Chitosan has a similar structure to cellulose. With a complex double helix structure, it is a linear polysaccharide after the removal of the acetyl group from chitin ((1,4)-2-acetamido-2-deoxy-β-d-glucose). Its molecular weight is often between 50 KDa and 2000 KDa. Chitosan is a highly abundant natural polymer material in which repeated monomer units are linked by covalent glycosidic bonds. The functional groups of chitosan include 6-position primary hydroxyl group, 3-position secondary hydroxyl group, and 2-position amino group or some *N*-acetylamino groups and glycosidic bonds. −NH_2_ and −OH in the chitosan molecule are chemically active, which can synthesize abundant chitosan derivatives via a reaction with a variety of chemical groups that are the main groups for chemical modification. However, the acetylamino bond and glycosidic bond are quite stable and not easy to break, which makes chemical modification difficult. C_3_-OH is a secondary hydroxyl group with great steric hindrance that cannot rotate freely and does not easily react. C_6_-OH is a primary hydroxyl group with small steric hindrance that can rotate freely. Therefore, C_6_-OH has greater activity than C_3_-OH. In addition, C_2_-NH_2_ generally has greater activity than C_6_-OH, so the reaction activity of the three is usually ranked as C_2_-NH_2_ > C_6_-OH > C_3_-OH [26,27]. A common problem in chitosan modification is that −OH or NH_2_ of chitosan will interfere with the reaction; thus, unwanted groups are introduced into the product. However, this problem can be avoided by introducing protective groups. Therefore, by changing the reaction conditions and reagents, chemical modification of chitosan can occur in the amino, hydroxyl, or amino and hydroxyl groups, thus forming *N*-modified, *O*-modified, or *N,O*-modified chitosan derivatives. In addition, the molecular weight and deacetylation degree of chitosan and its derivatives are closely related to their biological activity. Chitosan with a low molecular weight or high degree of deacetylation usually has stronger antitumor and antioxidant activities, whereas high molecular weight chitosan tends to have stronger antibacterial properties [27].

## 3. Chemical Modification Technology for Chitosan

### 3.1. Carboxylation Modification

Chitosan carboxylation refers to the process of introducing carboxyl groups into the molecule. The carboxylation reaction can occur with the hydroxyl or amino group on the chitosan molecule alone, or with the two active groups at the same time. The commonly used reagents for carboxylation modification of chitosan include glyoxylic acid and chloroacetic acid. When the reaction reagent is glyoxylic acid, glyoxylic acid can directly oxidize the hydroxyl groups on chitosan to carboxyl groups. However, when the reaction reagent is chloroacetic acid, chloroacetic acid can react with C_2_-NH_2_ and C_6_-NH_2_ on chitosan to generate *N,O*-carboxymethyl chitosan [28]. The water solubility, biocompatibility, and antibacterial properties of chitosan can be substantially improved by the introduction of carboxyl groups [29]. In addition, carboxylated chitosan, which has better flocculation, thickening, and film-forming properties than chitosan, has been widely used in medical, agricultural, and hygiene fields.

**Figure 1:**
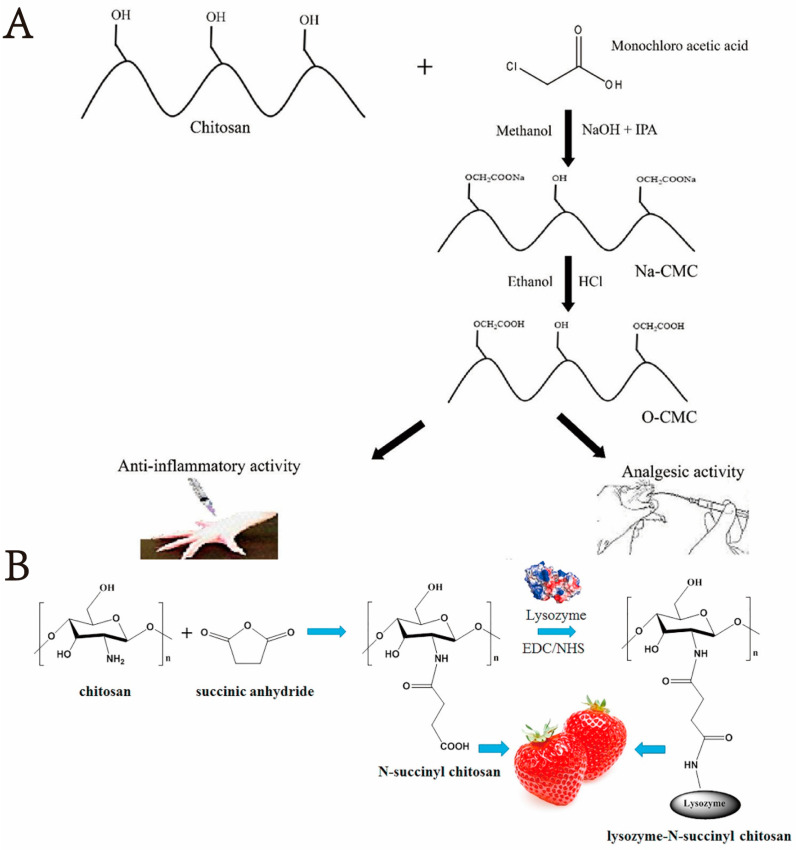
Carboxylation modification reaction scheme of chitosan: (**A**) Synthetic route of *O*-carboxymethylated chitosan. Adapted with permission from Ref. [34]. Copyright 2020 Elsevier. (**B**) synthetic route of lysozyme-*N*-succinyl chitosan. Adapted with permission from Ref. [36]. Copyright 2020 Elsevier.

At present, carboxymethyl chitosan is one of the most widely used chitosan derivatives. *O*-carboxymethyl chitosan has relatively good water solubility and pH sensitivity, which can be used in an intestinal targeted drug delivery carrier [30]. The order of carboxymethyl substitution in chitosan molecules is usually ranked as C_6_-OH > C_2_-NH_2_ > C_3_-OH, but the substitution position can be controlled by changing the reaction conditions and reagents. For example, to synthesize *O*-carboxymethyl chitosan, one can first add aldehyde so that the Schiff base reaction occurs and, therefore, protects the amino group in chitosan. Then, chloroalkane acid is added under alkaline conditions to induce *O*-carboxymethylation modification. Finally, the protective group is removed to obtain the target product [31,32]. In addition, *O*-carboxymethyl chitosan can be synthesized under a high alkali concentration (40 wt% NaOH) [33]. Adna et al. [34] used a simpler and more cost-effective method to synthesize *O*-carboxymethyl chitosan. The method was to first synthesize chitosan with chloroacetic acid, isopropanol, sodium hydroxide, and other reagents. Sodium carboxymethyl cellulose addition was followed by methanol and hydrochloric acid to synthesize *O*-carboxymethyl chitosan. the reaction scheme for which is shown in Figure 1A. It is worth noting that the newly discovered synthetic *O*-carboxymethyl chitosan has an analgesic effect. If the chitosan amino group is not protected, the reaction product is generally *N*,*O*-carboxymethylated chitosan [35].

Chitosan was acylated by succinic anhydride using lactic acid or dimethyl sulfoxide as the reaction medium, and *N*-carboxylated chitosan was synthesized by introducing the carboxyl group into the *N*-terminal of chitosan, the reaction scheme for which is shown in Figure 1B. For example, Niu et al. [36] synthesized a safe and non-toxic, antibacterial, fresh-keeping material. Under this method, chitosan first undergoes an acylation reaction with succinic anhydride to synthesize *N*-succinyl chitosan (NSC). Then, lysozyme (LSZ) is loaded into NSC to prepare lysozyme–NSC (LSZ–NSC), which reveals the influence of NSC on lysozyme activity and antibacterial activity. The results showed that compared with free lysozyme activity, lysozyme activity in LSZ-NSC was increased by 256%, and its antibacterial activity increased significantly under low concentrations. In addition, NSC and NSC-LSZ were used as antibacterial materials in strawberry preservation studies. The results showed that both NSC and NSC-LSZ extended the shelf life of strawberries. In particular, NSC-LSZ extended the shelf life of strawberries by 3 days. Environment.

The reaction site of carboxylation of chitosan is mainly C_6_-OH on the molecular chain of chitosan. The current method for synthesizing *O*-carboxylated chitosan is cumbersome, and it is necessary to introduce reagents such as aldehydes to protect C_2_-NH_2_. *O*-carboxylated chitosan has good solubility and has analgesic, antibacterial, antioxidant, anti-adhesion, and other activities [37], and the optimization of its reaction conditions should be emphasized in the future.

### 3.2. Alkylation Modification

Alkylation modification means part of the hydroxyl or amino groups of chitosan undergoes an alkylation reaction with halogenated hydrocarbons or sulfates to generate alkylated derivatives under alkaline conditions. Since C_2_-NH_2_ on the sugar molecule chain has a strong nucleophilic lone pair of electrons, the alkylation of chitosan usually occurs preferentially on C_2_-NH_2_. In addition to improving chitosan solubility, alkylated derivatives of chitosan also demonstrate good biocompatibility, coagulability, and antibacterial and hemostatic properties [38], which can be used for the preparation of medical gauze and adsorptive anionic surfactants.

**Figure 2:**
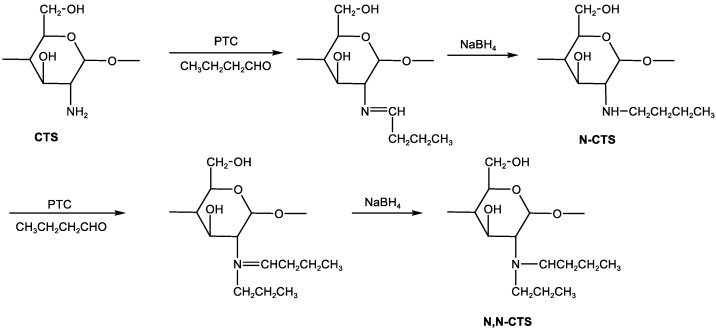
The reaction scheme of chitosan alkylation modification. The synthetic route of *N,N-*CTS [39].

Currently, *N*-alkylated chitosan derivatives are usually synthesized in two ways, namely by the Schiff base method or via a reaction with halogenated alkanes. The Schiff base method involves C_2_-NH_2_ on the chitosan molecular chain undergoing the Schiff base reaction with the aldehyde to form the Schiff base, and then *N*-alkylated chitosan is synthesized under the reduction of NaBH_4_. The reaction scheme is shown in Figure 2. For example, in a study by Jiang et al. [39], chitosan underwent a Schiff base reaction with *n-*butyraldehyde, and then alkylated chitosan was prepared through NaBH_4_ reduction, which then underwent a quaternization reaction with trichlorodihydroxypropyl triethyl ammonium chloride to produce *N*-alkylated quaternary ammonium chitosan. In the study, the minimal antimicrobial method was used to explore the antibacterial activity of the final product. It was found that the antibacterial rate of *N*-alkylated quaternary ammonium chitosan against *Escherichia coli* exceeded 99.9%. Chen et al. [40] used 4-octadecyloxybenzaldehyde and chitosan as raw materials to successfully connect the phenyloctadecyl group to the amino group on the chitosan molecular chain and transform it into an *N*-alkylated chitosan sponge (GACS). Studies have found that the combination of GACS and mesoporous silica nanoparticles with large pores can achieve a rapid bleeding control effect, which provides an excellent hemostatic agent in first aid. Huang et al. [41] also prepared alkylated chitosan via a Schiff base reaction between chitosan and lauric aldehyde under alkaline conditions and then characterized the grafted product. Later, the graft was prepared into a hydrophobic chitosan (HM-CHI) sponge. Fourier-transform infrared spectroscopy (FTIR) revealed that the dodecyl group was successfully grafted to the amino group on the chitosan backbone. The HM-CHI platelet agglutination experiment revealed that HM-CHI could accelerate the rate of platelet aggregation. In addition, HM-CHI can be used for hemostasis of femoral artery bleeding in rats. The hemostasis time was only 86.5 s. Compared with the hemostasis time of chitosan, the hemostasis rate increased significantly. The study suggests that HM-CHI should be used as a strong candidate for preparing safe and effective hemostatic materials. 

*N*-alkylated chitosan can also be prepared using halogenated alkanes. The introduction of an alkyl group of an appropriate molecular weight to the chitosan molecular chain will help improve chitosan solubility, but the introduction of too long a carbon chain will inhibit its solubility [42]. For example, Huo et al. [43] used the long-chain octyl group as the hydrophobic group and the glycol group as the hydrophilic group, which were respectively introduced into the chitosan molecular chain to synthesize amphiphilic chitosan (*N*-octyl-*O*-ethylene glycol chitosan). In addition, the alkyl chain length may affect the antibacterial activity of alkylated chitosan. For example, Sahariah et al. [44] developed a new method of efficiently modifying chitosan to synthesize a *N,N-*dialkyl chitosan derivative through reductive amination. The researcher also synthesized mono *N*-alkyl derivatives and quaternized them using optimized procedures. Studies found that these derivatives have antibacterial activity, and the activity size may be related to the alkyl chain length.

Since NH_2_ on the molecular chain of *O*-alkylated chitosan is not grafted, *O*-alkylated chitosan usually has relatively strong metal ion adsorption capacity. At present, it is difficult to synthesize *O*-alkylated chitosan, and there are few modification studies in this regard. The currently reported methods include the Schiff base method and the *N*-phthalylation method. The Schiff base method involves an initial reaction of C_2_-NH_2_ with aldehyde to form a Schiff base, then alkyl halide and C_6_-OH undergo and alkylation reaction, and finally the protective group is removed in the alkyd solution to obtain *O*-alkylated chitosan. The reaction scheme is shown in Figure 2. The bulky group formed by C_2_-NH_2_ of chitosan and the *N*-phthaloyl group boosts the selective reaction on C_6_-OH [45]. Hence, the *N*-phthalylation method is also used to first protect C_2_-NH_2_, and then the alkylation reaction is allowed, and finally the *N*-phthaloyl group with hydrazine is removed to obtain the product. However, Chen et al. [46] adopted another method and did not protect C_2_-NH_2_. Instead, he first designed an in situ reactor for the modification of chitosan *O*-alkylation, the derivatives of which were synthesized under reaction among chloroform, dodecanol, and *N,N*’-carbonyldiimidazole. The study showed that *O*-alkylated chitosan had significantly reduced crystallinity, which was easily dissolved in acetum and was thus suitable for in vitro cell transfection experiments.

At present, the modification technology of *N*-alkylated chitosan is relatively mature, and *N*-alkylated chitosan is also used in many fields. It is worth noting that the solubility of chitosan can be changed by changing the chain length of the alkyl group, so carbon chains of different lengths can be introduced separately to prepare amphiphilic chitosan, which may be a good direction for the application of alkylated chitosan in drug carriers.

### 3.3. Acylation Modification

Chitosan acylation products have good biocompatibility, drug-carrying capacity, and drug release control capability. Acylation modification of chitosan is a modification technology that presents certain challenges in chemical modification research. Organic acid derivatives (such as acid anhydrides, and acid halides) are used as acylating agents in a certain reaction medium, and then different aliphatic or aromatic acyl groups are introduced into the molecular chain of chitosan, which acylates chitosan. Acylated chitosan has good processing properties and slow-release effects [47]. The acylation reaction can induce *O*-acylation on OH to generate ester, *N*-acylation on NH_2_, or a simultaneous amino and hydroxyl product. Like the carboxylation reaction and alkylation reaction, the product and conditions of the acylation reaction are influenced by factors such as reagent types. Since C_2_-NH_2_ is highly active, acylation modification usually occurs preferentially on C_2_-NH_2_ to form *N*-acylated chitosan. *N*-acylated chitosan, which is easier to synthesize and can significantly improve the water solubility of chitosan, is usually used as a drug carrier or sustained-release agent in drug delivery. On the other hand, modification of *O*-acylated chitosan is more difficult and complicated to accomplish [48]; synthesized chitosan derivatives are fat-soluble and usually used for the preparation of polymer film materials to improve the hydrophobicity and stability of film materials. *N,O-*acylated chitosan is relatively easy to synthesize [49], However, its structure is quite complex and less used [48].

**Figure 3:**
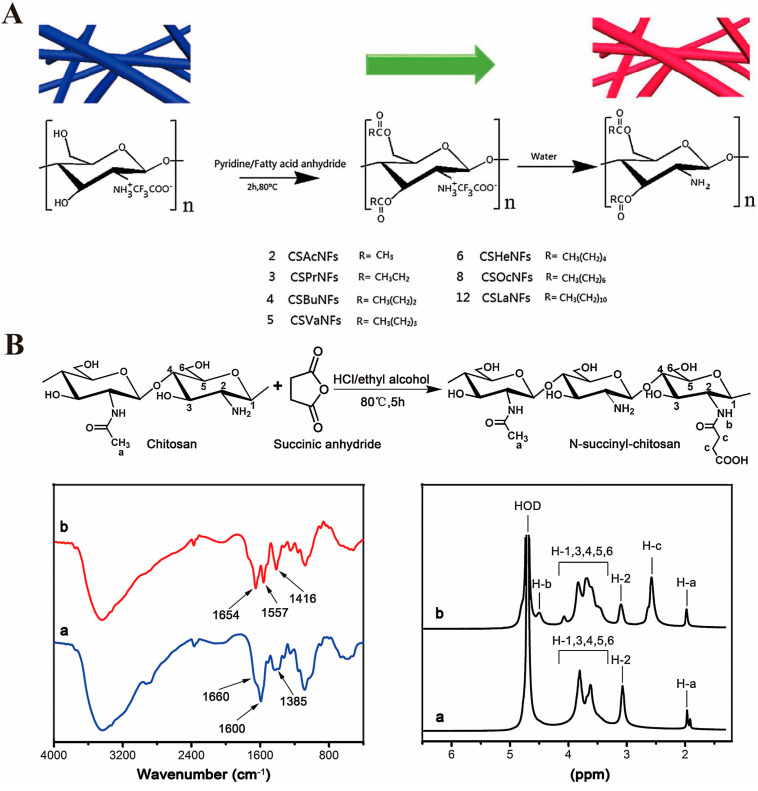
Modification scheme of *N*-acylated chitosan: (**A**) The synthetic route of *O*-acylated CSNFs. Adapted with permission from Ref. [50]. Copyright 2017 Elsevier. (**B**) the synthetic route of NSC and its structural characterization. Adapted with permission from Ref. [51]. Copyright 2016, Elsevier.

Since *O*-acylated chitosan is relatively difficult to prepare, it is usually necessary to add trifluoroacetic acid or methanesulfonic acid to protect C_2_-NH_2_ prior to the acylation reaction; then, the protective group must be removed after the reaction is complete. Zhang et al. [50] used pyridine as a catalyst, short-chain and long-chain fatty acid anhydrides as acylating agents, and chitosan nanofibers (CSNFs) as modifiers, and they synthesized *O*-acylated CSNFs under the presence of trifluoroacetate; its synthetic route is shown in Figure 3A. X-ray diffraction analysis revealed that *O*-acylation modification altered the crystal structure of CSNFs. In addition, scanning electron microscope analysis revealed that *O*-acylation modification can improve the fiber structure of CSNFs and increase their stability in humid environments.

Acylation modification of chitosan with acid anhydride can result in NSC with good solubility. For example, Tang et al. [51] synthesized NSC via acylation modification of chitosan using succinic anhydride, hydrochloric acid, and alkaline chitosan as raw materials; its structure was characterized by Fourier transform infrared spectroscopy, and hydrogen nuclear magnetic resonance spectroscopy was then used to investigate its solubility and toxicity, antibacterial properties, and ability to promote wound healing; its synthetic route and structural characterization are shown in Figure 3B. The results revealed that compared with chitosan, NSC had significantly higher solubility and better antibacterial activity. NSC significantly shortened the wound healing time and improved the action mechanism by which NSC promotes granulation tissue and enhances re-epithelialization capability. Due to the weak acylation characteristics of carboxylic acids, it is often necessary to activate the carboxyl group in the acylation reaction, usually via activating the reagent carbodiimide. For example, Ahmad et al. [52] prepared amphiphilic chitosan through a reaction of oleic acid and arginine with chitosan under carbodiimide activation and then self-assembled nanoparticles in an aqueous medium. Studies have demonstrated that the nanoparticles have significant antibacterial activity, especially against Gram-negative bacteria. The mechanism of action is to destroy the cell membrane of the bacteria, thereby effectively reducing the possibility of bacterial drug resistance.

The preparation method of *N*-acylated chitosan is simple, and the degree of substitution is high [53], which can significantly improve the solubility of chitosan and has achieved good results as a drug-carrying system. Therefore, when preparing hydrophilic or amphiphilic chitosan, *N*-acylation of chitosan can be an important selection direction when using nano-drug delivery systems. *O*-acylated chitosan is difficult to prepare and is less used. When hydrophobic modification of chitosan is required, it can be used as a modification direction.

### 3.4. Esterification Modification

Hydroxyl or amino groups on chitosan undergo an esterification reaction with oxygen-containing mineral acids or acid derivatives to produce esterified chitosan. Esterification modification enhances the adsorption and antibacterial abilities of chitosan to varying degrees [54,55,56].

**Figure 4:**
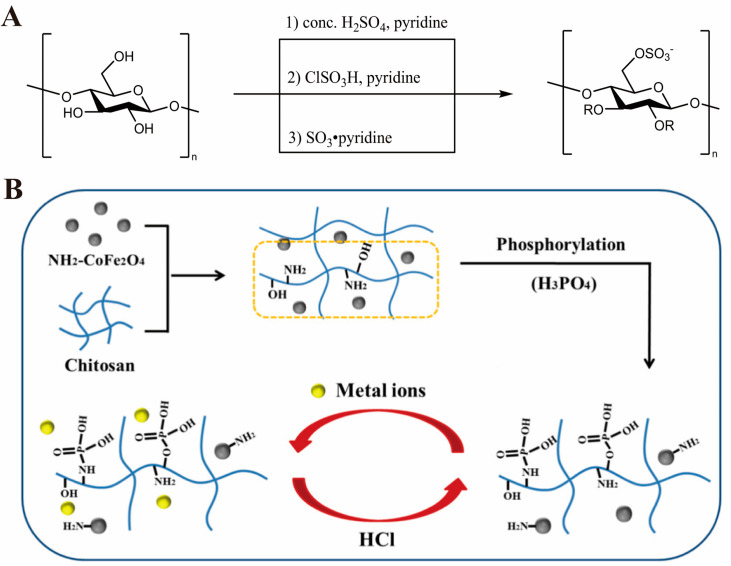
CS esterification modification scheme: (**A**) Synthesis route of sulfated chitosan. Adapted with permission from [66], Copyright © 2021 Elsevier. (**B**) synthesis route of phosphorylated chitosan. Adapted with permission from [65], Copyright © 2019 Elsevier.

Common esterification reactions include sulfation and phosphorylation. Sulfation involves obtaining an esterified product with a structure similar to heparin and that possesses excellent anticoagulant activity through a direct reaction of chitosan with sulfation reagents [56] (mainly including fuming sulfuric acid, sulfur dioxide, sulfur trioxide, and concentrated sulfuric acid), the modification scheme for which is shown in Figure 4A. The anticoagulant activity of sulfated chitosan is created via interactions between negatively charged sulfated groups and positively charged peptide sequences [57]. Sulfated chitosan can be used as a carrier for antiviral drugs. For example, Shazia et al. [58] esterified folic acid (FC) with chitosan (CS) to synthesize a CS–FC derivative and synthesized a BSA–CS–FC conjugate using bovine serum albumin nanoparticles. Its applicability to the diagnosis and treatment of tumors has been proven. Kazachenko et al. [59] proposed a new method for sulfating chitosan in 1,4-dioxane by putting 1,4-dioxane, sulfamic acid, and urea into a three-necked flask. The reaction was carried out in the flask under heating and vigorous stirring for 1–3 h. Wang et al. [60] used NaHSO_3_, NaNO_2_, *N*-succinyl, NaOH, and other reagents to synthesize *N*-succinyl chitosan sulfates under heating. The study found that compared with *N*-succinyl chitosan, *N*-succinyl chitosan sulfates have better anticoagulant effects, indicating that the introduction of sulfate groups can enhance the anticoagulant activity. Imran et al. [61] used a simpler and milder method to sulfate chitosan. They synthesized sulfated chitosan with reagents such as dimethylformamide, chlorosulphonic acid, and 20% NaOH, and they also confirmed that the introduction of sulfuric acid into chitosan groups increased their anticoagulant activity. Cao et al. [62] prepared nanoparticles based on 2-*N,*6-*O*-sulfated CS, and then loaded bone morphogenetic protei*N*-2 (BMP-2) on S-NPs to prepare BMP-2/S-NP nanomaterials, which were finally combined with gelatin sponge (G) to develop the composite implant (BMP-2/S-NP/G). Regarding the effects of critical size defects on vascularization and bone formation, the results showed that BMP-2/S-NP/G can significantly increase the formation of peripheral blood vessels and cardiovascular in rabbit radius and promote and accelerate bone regeneration.

Phosphorylation is usually employed to obtain a product with good water solubility via a reaction of methanesulfonic acid with CS, and the substitution site is mainly on the hydroxyl group of CS [63]. Wang et al. [64] first dissolved CS in methanesulfonic acid and then synthesized phosphorylated CS derivatives via a reaction with phosphorus pentoxide. Fourier-transform infrared spectroscopy (FTIR) and X-ray photoelectron spectroscopy were used to characterize the structure of the derivatives, FTIR analysis revealed that the phosphorylation reaction occurs in C_2_-OH of CS, because methanesulfonic acid protects the C_2_-NH_2_ of CS. Wu et al. [65] prepared a phosphorylated magnetic CS composite material (P-MCS) to adsorb the heavy metals lead and cadmium in an aqueous solution. The results showed that P-MCS had good adsorption against cadmium and lead, mainly due to ion exchange and direct complexation between heavy metal ions and functional groups. The study pointed out that P-MCS will eventually become an excellent source of material for treating wastewater containing heavy metal ions. Phosphorylated CS derivatives not only form an internationally recognized high-quality raw material for the synthesis of multifunctional plant growth regulators, but they have excellent heavy metal ion removal effects, the modification scheme for which is shown in Figure 4B. In addition, phosphorylated CS has bactericidal and metal chelating properties [55].

In addition to the modification of sulfated chitosan with sulfated reagents, many new methods have been added in recent years, but its application is still mainly focused on anticoagulation research. Sulfated chitosan is easier to synthesize, has better water solubility, and has better drug-carrying capacity. It can be considered to explore more activities and broaden its application range.

### 3.5. Sulfonation Modification

**Figure 5:**
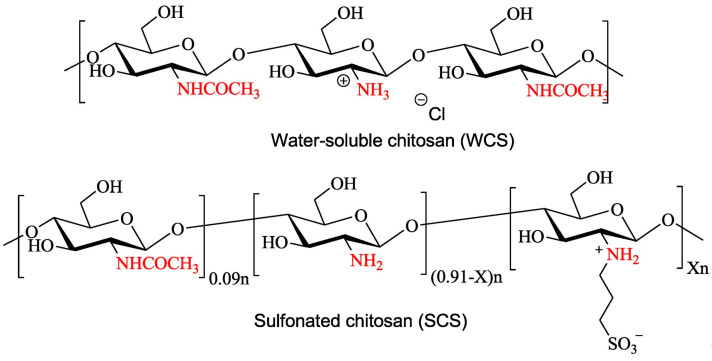
CS sulfonation modification scheme. Adapted with permission from Ref. [68]. Copyright 2019 Elsevier.

Sulfonation modification of CS involves introducing the sulfonate group into the reaction of hydroxyl or amino groups on the molecular chain of CS with concentrated sulfuric acid or sulphate, which preferentially occurs at the C_2_-NH_2_ of CS. Sulfonated CS has relatively strong antibacterial activity and anticoagulant activity [56,67]. For example, Huang et al. [68] prepared sulfonated CS (SCS) through a nucleophilic substitution reaction and explored the inhibitory activity of SCS against the adhesion and biofilm formation of *Escherichia coli* and *Staphylococcus aureus*, the modification scheme for which is shown in Figure 5. It was found that compared with unmodified CS hydrochloride, SCS has stronger stability and higher inhibitory activity against *E. coli* and *S. aureus* biofilm formation. The study also proposed that SCS can be used as an alternative for antibiotics and chemical protective agents in food and medical fields. Tsai et al. [69] synthesized sulfonated CS using CS and 1,3-propane sulfone as raw materials. After FTIR structural characterization and analysis, it was concluded that the sulfonation reaction took place in C_2_-NH_2_. Shenghua et al. [70] amidated CS and maleic anhydride and then sulfonated the product with sodium metabisulfite to synthesize sulfonated CS superplasticizer. FTIR characterization revealed that the sulfonation reaction occurred on CS C_2_-NH_2_. Studies have reported that sulfonated CS exhibits a similar molecular structure and performance as polycarboxylic acid superplasticizer, and the sulfonated CS water reducer also had a higher water reducing rate. The study further revealed that the trend to use renewable natural polymers (such as CS and its derivatives) and simple synthesis methods to synthesize high-performance superplasticizers are supported by evidence.

In addition, sulfo groups are often used as hydrophilic groups for amphiphilic CS derivatives. For example, Shelma et al. [71] introduced the hydrophilic sulfo group and hydrophobic lauroyl group to CS to prepare amphiphilic CS derivatives. In that study, sulfobenzoic acid cyclic anhydride was first reacted with CS to produce sulfonated CS, and then lauroyl chloride was added under certain conditions for acylation. After purification, lauroyl sulfated CS (LSCS) was obtained, and successful modification of CS was proven through FTIR and nuclear magnetic resonance (NMR). Studies have shown that LSCS can improve the blood compatibility of CS and significantly reduce chitosan-induced RBC aggregation and hemolysis.

At present, great progress has been made in the sulfonation of chitosan, and chitosan has been able to accurately control the reaction sites of sulfonated chitosan and the degree of substitution of sulfonate groups. Most importantly, sulfonated chitosan has rich biological activities, such as antiviral, antibacterial, anticoagulant, osteogenic, antioxidative, anticalcification, etc. [56], and more medical applications need to be focused on for the benefit of human health, which may be a good direction for the application of alkylated chitosan in drug carriers.

### 3.6. Quaternary Ammonium Salt Modification

The antibacterial properties of CS-quaternized derivatives and CS Schiff base derivatives are the most prominent among the numerous types of CS derivatives. The main purpose of quaternization of CS is to introduce quaternary ammonium groups or small molecular quaternary ammonium salts to the amino groups of CS, which can also occur on hydroxyl groups. Due to the high activity of amino groups, quaternary ammonium salt modification occurs preferentially on the amino group. Quaternary ammonium salt groups feature large steric hindrances, such as strong hydrate-ability, which significantly weakens the hydrogen bond between CS molecules added to the quaternary ammonium salt group, thereby significantly improving its water solubility. In addition, due to its permanent positive charge and large number of cations on the main chain, the CS derivatives exhibited excellent antibacterial activity and better antioxidant activity [72], which promotes CS application as a food preservative. It is worth noting that CS quaternary ammonium salt also achieved better biocompatibility, biodegradability, and good mucosal adhesion [73].

**Figure 6:**
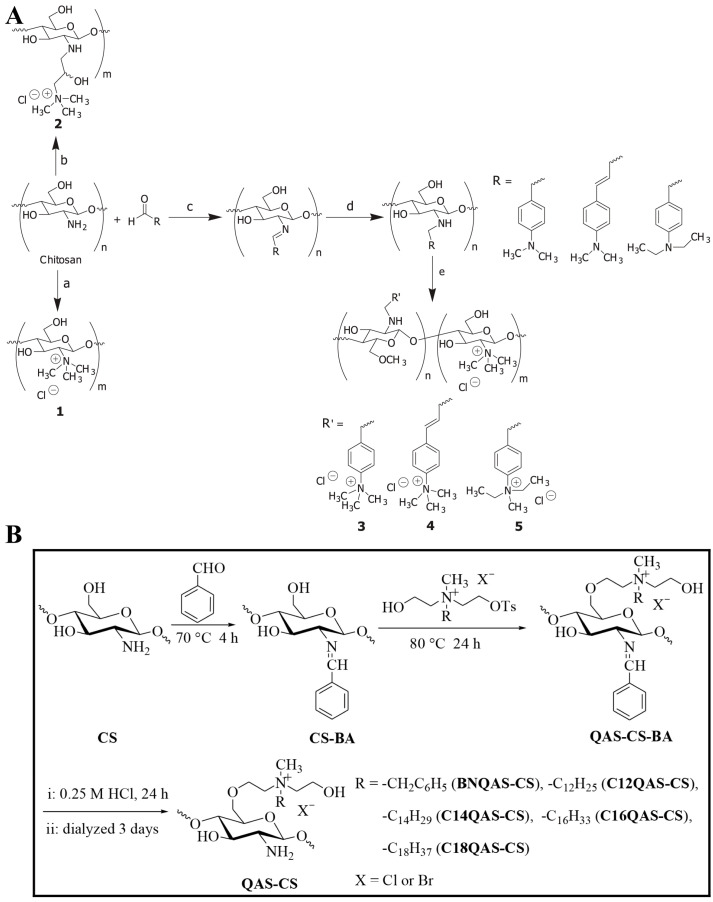
CS quaternary ammonium salt modification scheme: (**A**) The synthetic route of *N*-quaternary ammonium salt. Adapted with permission from [75], Copyright © 2012 Elsevier. (**B**) the synthetic route of *O*-quaternary ammonium salt. Adapted with permission from [80], Copyright © 2016 Elsevier.

There are two main synthetic methods for preparing *N*-quaternary ammonium CS derivatives. The first, which is the most common method, is to let excess halogenating reagents (such as alkyl iodide) directly react with CS to obtain halogenated CS quaternary ammonium salt. In terms of principles, this method utilizes the high reactivity of the alkyl iodide, making it possible to form a quaternary ammonium salt with an amino group. Wei et al. [74] obtained *N*-(2-pyridylmethyl), *N*-(3-pyridylmethyl), and *N*-(4-pyridylmethyl) through reactions among reagents such as pyridine carboxaldehyde, CS, and sodium. Then, by attacking secondary amine and *N*-pyridine with methyl iodide, the corresponding CS quaternary ammonium salt was obtained. The antioxidant activity results revealed that all diquaternary ammonium CS preparations achieved greater antioxidant activity than CS or monoquaternary ammonium CS. Similarly, Sajomsang et al. [75] used methyl iodide as a quaternization agent to quaternize *N*-(3-chloro-2-hydroxypropyl)trimethylammonium chloride with CS and thus prepared five kinds of quaternized CS derivatives; its modification scheme is shown in Figure 6A. This study evaluated its antifungal activity by using the inhibition zone method, minimum inhibitory concentration, and minimum fungicidal concentration. The results revealed that under neutral pH, quaternized CS exhibits antifungal activity against *Monascus*, *Trichophyton*, and gypsum molds.

The second method is to let quaternary ammonium-salt-containing alkylene oxide react with CS to obtain a quaternized chitosan-containing hydroxyl group. For example, Zhang et al. [76] quaternized 2,3-epoxypropyltrimethyl ammonium chloride with CS to synthesize 2-hydroxypropyltrimethyl ammonium chloride CS (HTCC) and then prepared *O*-methyl acrylamide quaternary ammonium salt of chitosan (NMA-HTCC) with active groups using HTCC and *N*-(hydroxymethyl)-acrylamide. Investigation of the antibacterial activities of CS and NMA-HTCC revealed that both CS and NMA-HTCC have an obvious antibacterial effect on *S. aureus* and *E. coli*, and NMA-HTCC has stronger antibacterial activity. Similarly, Zhang et al. [77] synthesized a new type of fiber-active CS derivative. The key step involved quaternization modification of CS and 2,3-epoxypropyltrimethyl ammonium chloride to produce a water-soluble CS derivative (i.e., HTCC). Next, the fiber-active CS derivative (i.e., NMA-HTCC) was synthesized through an HTCC reaction with *N*-(hydroxymethyl)-acrylamide. Evaluation of NMA-HTCC antibacterial activity against silk fabrics revealed that compared with the silk fabrics treated with CS and HTCC, silk fabrics treated with NMA-HTCC had significantly increased antibacterial effects against *S. aureus* and *E. coli*. In particular, the silk fabric had long-lasting antibacterial activity. Even after 50 repetitive scrubbings, the antibacterial rate of the silk fabric remained greater than 95%. In addition, studies have demonstrated that CS quaternary ammonium salt prepared via this method can be used as a carrier for hydrophilic drugs to achieve sustained and slow release [78]. Cele et al. [79] coupled different perfluorocarbon chains with CS via the Schiff base reaction and then synthesized a series of fluorinated quaternary ammonium CS derivatives through quaternization with glycidyl trimethyl ammonium chloride. Its antibacterial activity against a variety of clinical drug-resistant bacteria was investigated. The results demonstrate its rapid sterilization, which provides an alternative for patients who rely heavily on antibiotics.

*O*-quaternary ammonium CS is difficult to synthesize, and there are few studies that have investigated this issue regarding its synthesis. Similar to *N*-quaternary ammonium CS, *O*-quaternary ammonium CS has good water solubility and antibacterial activity. For example, a study that prepared five kinds of *O*-quaternary ammonium CS samples found that all *O*-quaternary ammonium CS preparations had significantly improved water solubility, all of which showed good antibacterial ability against Gram-positive bacteria [80]; the reaction scheme is shown in Figure 6B.

The modification of quaternized chitosan is cumbersome and time-consuming, so it is necessary to further explore a simpler and more efficient method to synthesize quaternary ammonium chitosan. Mainly used in antibacterial activity research, in order to broaden the application of quaternary ammonium chitosan, its role in other fields should also be investigated.

### 3.7. Graft Copolymerization Modification

CS molecules carry more active groups, which are prone to graft copolymerization reactions, and graft copolymerization reactions can occur on C_2_-NH_2_, C_3_-OH, and C_6_-OH [81]. Graft copolymerization is the most attractive method for improving physicochemical and biological properties through physical and chemical modifications of CS. It is also an important method for introducing CS polyfunctional groups (such as hydroxyl, carboxyl, ester, and amide). Properties of the final graft copolymerization product are mainly controlled by the molecular structure, length, and number of side chains. Graft copolymerization of CS mainly involves graft copolymerization with alcohols, esters, acids, and amides. Graft copolymerization modification not only improves the antibacterial, antitumor, antioxidant, and anticoagulant properties of CS, but it is also applicable to fabrics and cellulose surface modification.

Common graft copolymerization methods include oxidative coupling copolymerization, free radical graft copolymerization, and condensation copolymerization, among others. Li et al. [82] prepared chitosan–ferulic acid (CS–FA) derivatives through a carbodiimide-mediated coupling reaction and used the derivatives as a carrier to load bovine serum albumin onto it, thereby protecting and controlling the slow release of the drug. Then, the thermal performance of CS–FA was evaluated by thermogravimetric analysis and differential scanning calorimetry. The results show that compared with chitosan, CS–FA derivatives had low crystallinity and high thermal stability. In addition, CS–FA had an obvious swelling rate and good in vitro sustained-release performance. Wang et al. [83] also used the carbodiimide coupling method to graft CS with phenolic acids, such as gallic acid (GA) and caffeic acid (CA), and then characterized the structure and properties of the graft. Afterwards, the grafts were formed into composite films by tape casting, and the antioxidation and antibacterial activities of the films were characterized. Infrared and NMR spectroscopy analyses of the grafts revealed that GA and CA were successfully grafted on CS C_2_-NH_2_. The antioxidant activity results showed that compared with the CS film, the GA-g-CS and CA-g-CS films had enhanced antioxidant activity. The antibacterial activity results showed that compared with the CS film, the CA-g-CS film had a significantly stronger inhibitory effect on *Bacillus subtilis* (g+) and *S. aureus* (g+) (*p* < 0.05).

**Figure 7:**
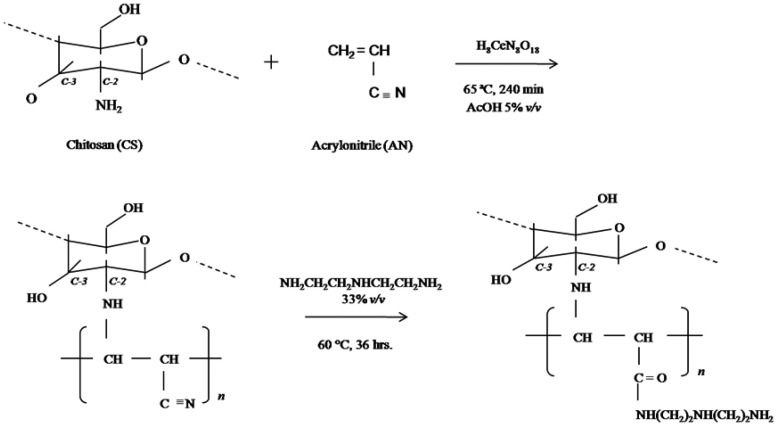
APANCS synthetic route [84].

The reagents used in free radical graft copolymerization are relatively safe and constitute commonly used graft copolymerization modification reaction systems. For example, Ja Dena-Aguilar et al. [84] used acrylonitrile (AN), CS, and cerium ammonium nitrate (CAN) as initiators to synthesize polyacrylonitrile–graft–chitosan (PAN–g–CS) copolymer and then reacted it with diethylenetriamine to synthesize polyacrylonitrile–graft–chitosan (APANCS) aminated copolymer. The research shows that APANCS can better adsorb Pb^2+^, Cd^2+^, Zn^2+^, and other heavy metal ions in water, the synthetic route of which is shown in Figure 7. Guo et al. [85] used hydrogen peroxide and ascorbic acid as free radical mediators to graft CA onto CS. Fourier infrared spectroscopy and NMR analysis proved that CA was grafted onto the CS amino group. An antioxidant performance test revealed that compared with CS and CA, chitosan–CA derivatives possess stronger antioxidant activity, and the study further found that chitosan–CA derivatives can be used as a new type of bifunctional surfactant for polymers. In another study, free radical graft copolymerization was used to graft CS and 2-hydroxyethyl acrylate, and then the product was prepared into a sponge dressing. Examination of its swelling ability, biocompatibility, and antibacterial activity revealed that the dressing had significant antibacterial effects on both *Pseudomonas aeruginosa* and *S. aureus* [86].

In general, the synthesis method of chitosan graft copolymerization has diversity. It can introduce various groups, which is an important direction for the diversification of chitosan derivative materials and functions. In the future, we can focus on grafting drugs with some better therapeutic effects, which may give stronger and more efficacy to chitosan.

### 3.8. Schiff Base Reaction

The Schiff base reaction of CS involves a condensation reaction of the amino groups on CS with carbonyl compounds (such as fatty aldehydes, aromatic aldehydes, and ketones) in a neutral medium to remove water molecules and synthesize the Schiff base with imine group characteristics. Commonly used solvent media for the CS Schiff base reaction include acetic acid, ethanol, and methanol, or their mixtures. On the one hand, Schiff base modification of CS changes the molecular structure of the CS molecular chain and increases the number of positively charged ions, which significantly strengthens the water solubility and antibacterial activity of CS. Schiff base modification of CS is, therefore, widely used to enhance the antibacterial properties of CS. On the other hand, the Schiff base reaction of CS is often used to protect amino groups on CS, so CS modification only occurs on the hydroxyl groups.

**Figure 8:**
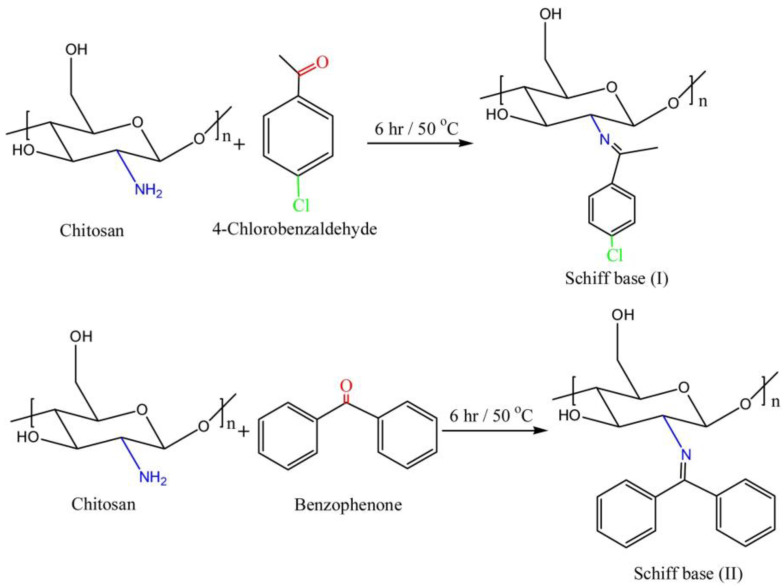
CS Schiff base modification scheme. Adapted with permission from Ref. [87]. Copyright 2016 Elsevier.

The CS Schiff base has extensive biological activities, such as antibacterial, antitumor, and antioxidation properties, and can be used as a catalyst. For example, Tamer M. Tamer et al. [87] prepared two aromatic CS Schiff bases with 4-chlorobenzaldehyde or benzophenone as a reactant to improve the antibacterial properties of CS, and its modification scheme is shown in Figure 8. The antibacterial test of two CS derivatives revealed that for most microorganisms, the two products had significantly higher antibacterial activity than CS, which could be applied to the preparation of antibacterial wound dressings. Nadia Q et al. [88] used 2-chloroquinoline-3-carbaldehyde, quinazoline-6-carbaldehyde, and oxazole-4-carbaldehyde as modification reagents and synthesized three new types of CS Schiff base derivatives through a Schiff base reaction with CS. ^1^H and ^13^C NMR and FTIR spectroscopy confirmed the successful reaction between the modified reagent and CS C_2_-NH_2_. The inhibition zone method was used to investigate the antibacterial activity of synthetic compounds against Gram-negative and Gram-positive bacteria, and the 3-(4,5-dimethylthiazol-2-yl)-2,5-diphenyltetrazolium bromide (MTT) method was used to evaluate the cytotoxicity of synthetic compounds. The antibacterial activity results revealed that compared with CS, CS Schiff base derivatives achieved significantly increased antibacterial activity against fungi and bacteria. The MTT results revealed that the synthesized compounds were not cytotoxic, which suggested that they can be used as new nontoxic biomaterial candidates for enhancing antibacterial properties.

At present, the preparation method of chitosan Schiff bases is relatively mature, and they can be easily and simply synthesized. Chitosan Schiff bases generally have a wide range of biological activities, so chitosan can be reacted with different aldehydes or ketones to synthesize more different chitosan Schiff base derivatives to develop more functional materials with good pharmaceutical effects.

Table 1 provides a comprehensive evaluation of the preparation methods and effects of the above-mentioned chemical modification technologies.

The conditions of different chemical modification techniques of chitosan and the products obtained are shown in Table 2.

## 4. The Main Activities of CS and Its Chemically Modified Derivatives

Chemically modified derivatives of CS also have extensive activities, the most researched of which are improved adsorption, antibacterial, antitumor, hemostasis, and antioxidant activities. Since CS derivatives have the same basic skeleton as CS, the mechanism of action of its various activities is similar to that of CS. At present, the exact mechanisms of various activities of CS are not fully understood. The following sections provide an overview of the mechanisms of the main activities of CS.

### 4.1. Antibacterial Activity

At present, numerous studies have shown that CS has broad-spectrum antibacterial activity, and due to its rich resources, it is considered to be one of the most ideal antibacterial materials. CS forms a layer of polymer film mainly through adsorption on the cell surface to cut off the channel of nutrient transportation into the cell, thereby achieving antibacterial and sterilization effects. Other studies have shown that because CS is positively charged, it can attract and combine with negative charges on the surface of the bacterial cell wall to induce bacterial cell wall lysis [90,91]. The antibacterial activity of CS is correlated to internal factors, such as its molecular weight, degree of deacetylation, and concentration, as well as external factors, such as pH and temperature [9]. However, the amino group of CS has a weak positive charge, so the antibacterial activity is too weak to meet antibacterial needs in various fields. In addition to altering internal factors and external conditions of CS, CS can also be chemically modified to introduce more positively charged groups for improved antibacterial activity.

Like CS, chemically modified derivatives of CS usually have antibacterial activity. Quaternary ammonium CS, carboxylated CS, hydroxylated CS, and hydrophobic alkylation CS have outstanding antibacterial properties. For example, Jin et al. [92] synthesized CS Schiff base derivatives with CS and citral under high-intensity ultrasound. Compared with CS, the antibacterial activity of CS and Schiff base against *E. coli, S. aureus, and Aspergillus niger*, CS Schiff base derivatives have more significant antibacterial activity, which is concentration-dependent. In addition, Dragostin et al. [93] synthesized six chitosan-sulfonamide derivatives, whose antibacterial activity tests showed that its antibacterial and antifungal properties were significantly improved in comparison with CS. In addition, the swelling, biodegradation rates, and healing effect on injured rats were improved. The study demonstrated that these chitosan-sulfonamide derivatives can be applied to the preparation of novel wound dressings, especially for burn wounds. Li et al. [94] synthesized an *O*-quaternary ammonium *N*-acyl thiourea CS with dual antibacterial groups, and its antibacterial activity test proved its broad-spectrum antibacterial activity. In addition, integrity analysis of the cell membrane and transmission electron microscopy tests found that the mechanism of action was to destroy the bacterial cell membrane. Furthermore, the introduced quaternary ammonium and thiourea groups increased the positive charge of the CS derivative, thereby enhancing its antibacterial activity.

The antibacterial ability of chitosan derivatives is related to the number of positively charged groups on the molecular chain, so it is possible to focus on the simultaneous modification of the amino group and hydroxyl group of chitosan, and it is possible to obtain chitosan derivatives with high antibacterial activity for developing a new generation of antibacterial agents.

### 4.2. Antitumor Activity

CS has a positive charge, so it can act directly on tumor cells, which possess more negative charges than normal cells, thereby inhibiting transfer and growth and interfering with metabolism, thereby inducing apoptosis in malignant tumors [95]. In addition, CS can increase the activity of macrophages and enhance the body’s immune function [96,97,98]. CS can attach to vascular endothelial adhesion molecules, inhibit the formation of vascular endothelial cells, and prevent cancer tissues from infiltrating, spreading, and transferring to surrounding tissues, thereby achieving antitumor effects [99,100].

**Figure 9:**
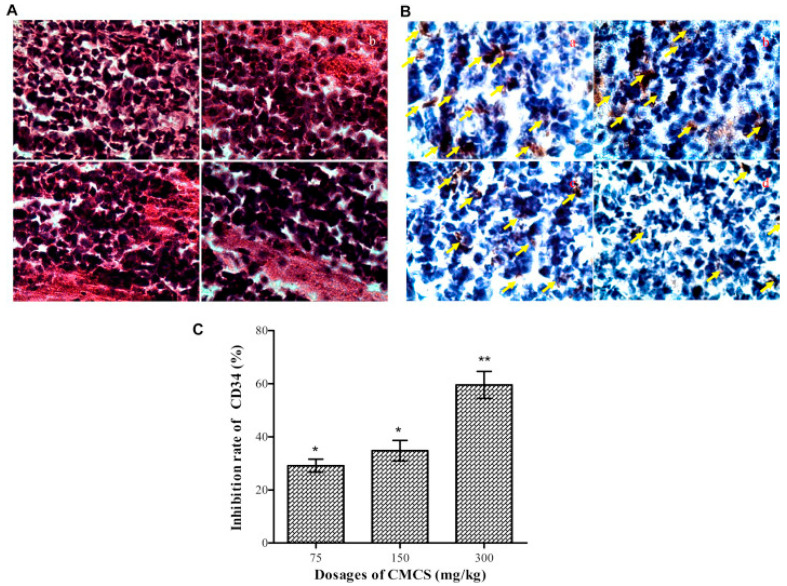
Effects of CMCS on histopathology and CD34 expression in H22 tumor tissue. (**A**) Effect of the CMCS on histopathology of H22 tumor tissue was recorded by the light microscope. (**B**) Photographs of effect on CD34 expression of H22 tumor tissue were taken by the light microscope. (**C**) Inhibition rate of CMCS on CD34 expression of H22 tumor tissue, * *p* < 0.05, ** *p* < 0.01 significant difference compared with control group. Adapted with permission from Ref. [102]. Copyright 2015 Elsevier.

Some chemically modified derivatives of CS also have antitumor activity, the more prominent of which are quaternary ammonium, sulfation, carboxylation, and graft copolymerization derivatives. Lee et al. [101] grafted CA (i.e., CFA) to the amino group of CS to synthesize a chitosan–CA derivative (ChitoCFA), which is an anticancer compound, and compared the anticancer effects of CFA and ChitoCFA in CT26 colon cancer cells. The results revealed that compared with CFA, ChitoCFA may accelerate the apoptosis rate of tumor cells. In addition, the study also found that ChitoCFA has a good antiproliferation effects on tumor cells and is a promising anticancer compound. In addition to having strong water solubility, studies found that carboxymethyl CS (CMCS) inhibited the growth of liver cancer tissues in mice and promoted the necrosis of tumor cells. The mechanism of action is that CMCS inhibits tumor vessel growth and enhances the body’s immune function. Jiang et al. [102] evaluated the antitumor metastasis effect of carboxymethyl CS on liver tumors, finding that carboxymethyl CS can significantly inhibit tumor cell migration; its anti-tumor results are shown in Figure 9. In addition, Jiang et al. [103] synthesized SCS by introducing a sulfate radical to the CS molecular chain and then synthesized sulfated benzaldehyde CS (SBCS) by introducing phenyl groups to SCS. Studies have found that both SCS and SBCS can significantly inhibit the proliferation of MCF-7 cells and induce its apoptosis. In addition, compared with SCS, SBCS has better antitumor effects and lower IC50.

Overall, chitosan derivatives have weak antitumor effects and can be used for adjuvant therapy of cancer. We can use compounds with more positive charges or drugs with certain anti-tumor effects to modify chitosan to synthesize chitosan derivatives with anti-tumor effects. Then using chitosan derivatives as drug carriers to deliver anticancer drugs may have better and longer-lasting efficacy.

### 4.3. Hemostatic Activity

CS is a new type of polymer hemostatic agent that can stop bleeding through multiple methods. Because CS is noncytotoxic and has good biocompatibility, antibacterial activity, and hemostatic properties, it has been used clinically as a hemostatic candidate. The anticoagulant effect of CS is mainly related to its structural parameters, such as the deacetylation degree, molecular weight, and substituents [10]. There are three main aspects of the hemostatic mechanism of chitosan. First, CS has a positive charge, whereas red blood cells have a negative charge. The two adhere and aggregate and then undergo an electrostatic neutralization reaction, which causes repulsion to dissipate, leading to blood coagulation [104,105,106]. Second, CS quickly promotes platelet thrombosis, thereby accelerating blood coagulation [107]. Third, CS can bind to plasma proteins and some important coagulation factors in vivo, resulting in firmer blood clots [108].

**Figure 10:**
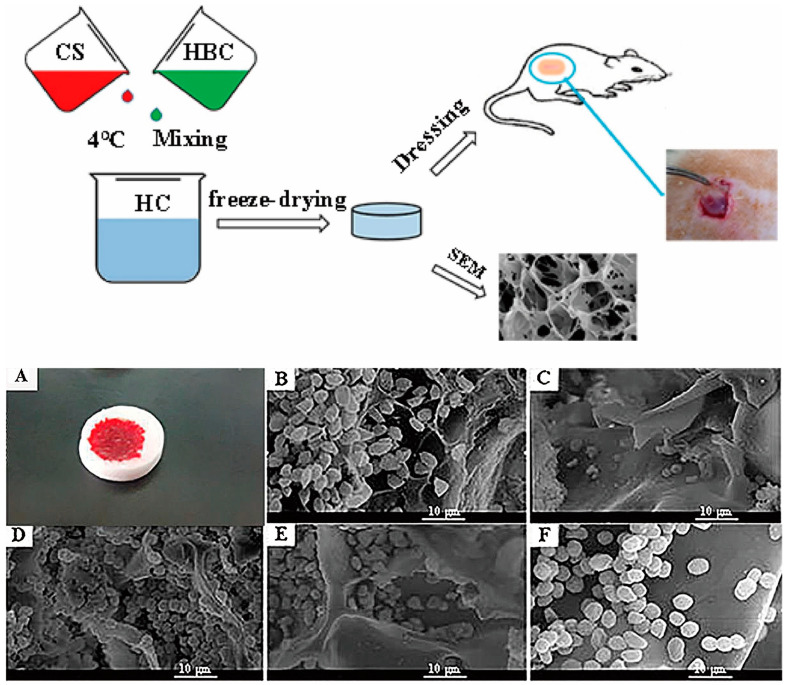
Preparation of composite sponge and its hemostatic test result. (**A**) The photo of the composite sponge with blood; (**B**–**F**) The SEM images of blood cells adhesion of the CS, Hydroxybutyl chitosan (HBC), HC-1 (M_CS_:M_HBC_ = 1:3), HC-2 (M_CS_:M_HBC_ = 1:2) and HC-3 (M_CS_:M_HBC_ = 1:1) respectively. Adapted with permission from Ref. [109]. Copyright 2018 Elsevier.

CS derivatives with outstanding hemostatic properties include alkylated CS, carboxylated CS, and quaternary ammonium CS. For example, Hu et al. [109] incorporated chitosan into hydroxybutyl chitosan and prepared a composite sponge by freeze-drying. The study found that the composite sponge had higher porosity than chitosan sponge and hydroxybutyl chitosan sponge (about 85%), greater water absorption (about 25 times), better flexibility, and lower coagulation index, and its whole blood coagulation and blood cell adhesion tests show that the composite sponge can absorb more red cells permeated all over the porous structure. In the structure, blood can also form a viscous gel to promote blood coagulation due to the interaction between cationic CS and negatively charged blood cells; its preparation method and hemostasis test are shown in Figure 10. Zhao et al. [110] prepared *N,O-*CMCSs with different degrees of substitution and examined the hemostatic performance using the dynamic clotting time method and the in vivo coagulation test. The results showed that CMCSs showed excellent hemostatic effects under the substitution degree of 60–80% but had decreased hemostatic ability under a high degree of substitution. The reason could be that the high level of carboxymethylation increases the negative charge of CS, thus causing CS to lose the ability to attract red blood cells and aggregate platelets. To improve the hemostatic properties of CS nonwoven fabrics, Yan et al. [111] introduced succinyl groups (NS), carboxymethyl groups (CM), and quaternary ammonium groups (TM) into CS nonwoven fabrics to synthesize NSCS, CMCS, and TMCS nonwoven fabrics. Hemostatic performance evaluation revealed that the three had strong hemostatic effects, of which NSCS1 nonwoven fabric had the shortest hemostatic time (147 ± 3.7 s) and the lowest level of blood loss (0.23 ± 0.05 mL). Exploration of the blood coagulation mechanism found that SCS and CMCS nonwoven fabrics could activate the intrinsic pathway of blood coagulation to accelerate blood coagulation.

In recent years, research on hemostasis of chitosan and its derivatives has gradually increased, arousing great interest of researchers. In addition to developing more chitosan derivative hemostatic materials, chitosan and its derivatives can also be mixed with other hemostatic materials to prepare composite hemostatic materials to achieve rapid and effective hemostasis.

### 4.4. Antioxidant Activity

The free radical reaction has been considered to be the pathogenesis of several specific diseases in humans, such as cancer; heart, cardiovascular, and cerebrovascular diseases; and Alzheimer’s disease. Free radicals, which have extremely high oxidative activity and are extremely unstable, can attack cell membranes, mitochondrial membranes, and unsaturated fatty acids in membranes, thus resulting in enhanced lipid peroxidation. The amino group and two hydroxyl groups of CS have a strong hydrogen supply capacity, which can react with free radicals to develop a scavenging capacity [112]. In addition, CS can significantly reduce serum-free fatty acid and malondialdehyde concentrations and increase antioxidant enzyme activities, such as superoxide dismutase, catalase enzyme activities, etc., thereby scavenging free radicals. Studies have found that the antioxidant activity of CS mainly depends on the molecular weight. Its antioxidant activity is stronger with a lower molecular weight, whereas CS with a high molecular weight has no antioxidant activity against superoxide and hydroxyl radicals [113]. Common CS derivatives with relatively strong antioxidant activity include quaternized CS and graft copolymers containing polyphenol compounds.

**Figure 11:**
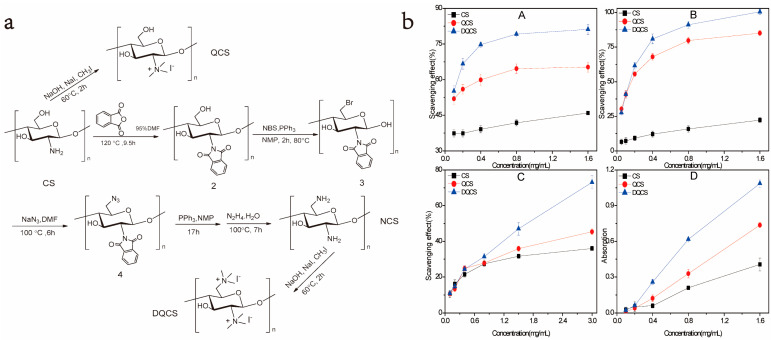
Preparation method of QCS and DQCS and test results of antioxidant activity. (**a**) Synthetic route for the preparation of QCS and DQCS; (**b**) Antioxidant effect of samples [114].

In addition to excellent antibacterial activity, quaternized CS also has relatively strong antioxidant activity. For example, Luan et al. [114] prepared mono-*N*-quaternized (QCS) and double-*N*-diquaternized (DQCS) chitosan derivatives and then evaluated the antioxidant activity of CS, QCS, and DQCS from two indicators, namely, free radical scavenging ability and iron reduction ability, and its preparation method and antioxidant activity test results are shown in Figure 11. The results revealed that the antioxidant capacity of the three was ranked as CS < QCS < DQCS, which indicates that adding quaternized groups to the chitosan molecular chain can enhance the antioxidant activity of chitosan. In addition, Skorik et al. [53] synthesized succinyl CS (SC) derivatives and glutaryl CS (GC) derivatives with specific degrees of substitution. The study found that both SC and GC exhibited significant and similar antioxidant activity, which increased with the increasing degree of substitution. In addition, the study also found that SC and GC have good anticoagulant activity. As a safe material with antibacterial, antitumor, anti-inflammatory, antidiabetic, and strong antioxidant activities [115], polyphenol has been widely used in food manufacturing, pharmaceuticals, and medicine. After CS is grafted with a polyphenol compound, the antioxidant activity will be significantly improved due to the synergistic effect of CS and polyphenol. For example, Liu et al. [116] grafted CA and ferulic acid (FA) to CS molecular chains to synthesize two phenolic acid–CS copolymers. An in vitro antioxidant test revealed that the antioxidant activity of two phenolic acid–CS copolymers and CS is ranked as CA–g–chitosan > FA–g–chitosan > CS. In addition, an in vivo antioxidant activity test showed that the two phenolic acid–CS copolymers could significantly increase antioxidant enzyme activity and reduce the level of malondialdehyde in the serum and liver of D-galactose-induced aging mice, so that the two phenolic acid–CS copolymers had much greater antioxidant activity in vivo than CS.

In recent years, the preparation of conjugates by grafting chitosan with different antioxidants has received considerable attention. This study can not only improve the instability, volatility, and easy degradation of antioxidants but also improve the antioxidant capacity of chitosan. In addition to studying the antioxidant activity of chitosan and its derivatives, its antitumor activity should also be studied, because the ability of chitosan derivatives to scavenge free radicals can enhance their antitumor activity [117]. 

Table 3 summarizes the studies on antibacterial, antitumor, hemostatic, and antioxidant activities of chitosan derivatives.

## 5. Main Applications of CS and Its Chemically Modified Derivatives

### 5.1. Textile Industry

In recent years, there has been a substantial increase in the number of studies and applications that utilize CS to modify the surface of textiles to improve its performance. CS plays antibacterial, thickening, blood coagulation, deodorization, antistatic, antiwrinkle, and wound healing roles in the surface modification of textiles [16,118]. In addition, CS can enhance the color depth of textiles and serves as a good auxiliary for printing paste [119]. However, CS has relatively low solubility under neutral or alkaline conditions, which leads to its weak binding to textile fibers and poor washing durability [120,121]. CS derivatives can improve CS solubility and its ability to bind different textile fibers, thus solving chitosan-related problems in the textile industry. The application of common chitosan derivatives in the textile industry is shown in Table 4.

CS and its derivatives are usually used for antibacterial treatment in the textile industry. For example, Gupta et al. [122] used a water-soluble carboxymethyl CS in cotton fabrics through a dry nipping and curing process. Assessment of the antibacterial activity and washing durability of the cotton fabric revealed that the cotton fabric had obvious antibacterial activity against *S. aureus* and *E. coli* and improved the washing durability. It is well known that both quaternary ammonium salt groups and Schiff bases have relatively strong antibacterial activity. Fu et al. [123] used 2,3-epoxypropyltrimethyl ammonium chloride and benzaldehyde as a modifier to enhance the antibacterial activity of textiles, synthesized *O*-type quaternized-*N*-CS Schiff base (*O*-QCTSS) through quaternary ammonium modification and Schiff base modification. The study found that the *O*-QCTSS-treated cotton fabric had strong antibacterial activity and excellent washing durability. Even after 20 consecutive washings, the antibacterial activity of the cotton fabric remained above 75%.

Because chitosan and its derivatives have a wide range of antibacterial activities, they are mainly developed into antibacterial fabrics, wound accessories, sutures, and other products for the textile industry. Attention should also be paid to its research and development in sportswear, printed fabrics, and anti-odor fabrics.

**Table 4:** Application of chitosan derivatives in the textile industry.

Name	Combine with Textiles	Application	Effect	References
*O*-Acrylamidomethyl-*N*-[(2-hydroxy-3-dimethyldodecylammonium) propyl] chitosan chloride	Form covalent bonding with cellulosic fibers	Cotton fabric	Showing durable antimicrobial functions even after 30 consecutive home launderings, showed improved uptakes, fixation rates, K/S values, and fastness of reactive dyes without using auxiliary salt	[124]
*O*-Methyl acrylamide quaternary ammonium salt of chitosan	Form covalent bonds with cellulose fiber	Cotton fabric	Demonstrating excellent durable wrinkle-resistance and antibacterial activity against *Staphylococcus aureus* and *Escherichia coli*	[125]
Carboxymethyl chitosan	Anchored to the surface of cotton fiber via esterification	Cotton fabric	Activity against *S. aureus* and *E. coli* was above 99.9%, the evaluation of water absorption and flexibility Showed that the modified cotton fabrics were safe and comfortable	[126]
Chitosan-based water-dispersible polyurethane	Using pad-dry-cure procedures	Polyester/cotton textiles	Improvement in the antibacterial activity	[127]
Carboxymethyl chitosan	Pad-dry-cure method	Cotton gauze	Promising to be used as bacterial filter	[128]
Carboxymethyl chitosan	The coating process	Gauze	Have antifungal activity	[29]
Ammonium-salicylidene chitosan Schiff base	Pad-dry-cure method	Cotton fabrics	The treated cotton fibers demonstrated strong and sustainable antimicrobial impacts on *S. aureus*, *E. coli*, and *C. albicans* pathogens	[129]
Nanocomposite based on silver nanoparticles and carboxymethyl chitosan	Incorporation	Cotton	The functionalized fabric showed 100% antibacterial activity against *E. coli* and *S. aureus* and good antifungal activity against *C. albicans* and *A. niger*	[130]
Chitosan-acrylamide (Ch-Ac)	Graft	Wool	Antibacterial and antioxidant activities of Ch-Ac-treated wool yarns were significantly improved	[131]
Novel chitosan-based polymeric dye	Padding	Cotton	The dyed cotton showed an outstanding mosquito repellency (100%) with good durability	[132]

### 5.2. Wound Healing

Skin wound treatment usually refers to hemostasis and anti-inflammatory treatment. Inappropriate wound treatment can cause serious complications and loss of function. CS and its derivatives demonstrate excellent properties, such as biocompatibility, osteoconduction, and a porous structure, and it promotes cell growth, hemostasis, broad-spectrum antibacterial activity, and tissue adhesion, which can be used as a wound healing accelerant [133,134,135] and are widely prepared into different forms of anticoagulant materials such as sponges, hydrogels, particles, bandages, and dressings [136,137,138]. When acting on the wound, these CS sponges, dressings, or particles can combine with tissue fluid exuded from injured skin to form a CS hydrogel, thereby promoting healing. CS can accelerate wound healing in each stage to some extent. For example, in the early stage of the healing phase, CS can exert its hemostatic effect, which promotes the infiltration and migration of neutrophils and macrophages [139], thereby promoting the formation of granulation tissue. For example, Pandini et al. [134] prepared chitosan film biomaterials, and then filled them in the experimental defect nasal bones of long-term alcoholic and non-alcoholic rats, respectively, and found that chitosan film caused alcoholism. Mice and hangover rats have good biocompatibility and can promote the proliferation of osteoblasts in the damaged nasal bone of rats, and achieve the effect of promoting osteogenesis.

Due to poor water solubility and weak antibacterial activity, CS has limited application in wound healing. CS derivatives can improve the antibacterial activity of CS and enable more and stronger activity, which effectively expands the application scope of CS in the field of wound healing. The application of common chitosan derivatives in wound healing is shown in Table 5. For example, Zheng et al. [140] coated the surface of cotton gauze with carboxymethyl chitosan–gelatin–alginate to develop a fast hemostatic composite dressing. The composite dressing achieved relatively strong liquid absorption, excellent biocompatibility, and blood compatibility. More importantly, compared with cotton gauze, the composite dressing demonstrated a shorter hemostasis time and less blood loss in the mouse liver injury model and mouse tail amputation model. Moreover, the dressing could also promote wound surface healing. Sulfonamides are effective drugs for the prevention and treatment of bacterial infections. To improve the antibacterial activity of wound dressings, Dragostin et al. [93] introduced sulfadiazine, sulfadimethoxine, and sulfamethoxazole to CS and then synthesized three chitosan-sulfonamide derivatives. Evaluation of the biodegradation behavior of the three chitosan-sulfonamide derivatives in vivo revealed that they effectively alleviated the wound swelling and achieved a higher biodegradation rate. In the rat burn wound model test, the three chitosan-sulfonamide derivatives had a significantly better effect than CS in promoting the healing of rat burn wounds. 

Chitosan and its derivatives have good biocompatibility and hemostatic, antibacterial, and wound healing properties, which have aroused great interest in the use of chitosan derivatives for wound healing in recent years. Many dressings and drugs related to chitosan and its derivatives have been successfully developed and have achieved good efficacy, but there are few clinical trials on these drugs, which should be strengthened to truly benefit human health.

### 5.3. Drug Carrier

The amino group of CS is positively charged and interacts electrostatically with the negative charge on the mucosal surface to create a mucosal adhesion effect [151,152]. Generally, mucosal adhesion properties will increase the adhesion time of the drug in the body’s absorption site, which helps control drug release, improve drug absorption, and enhance the drug therapeutic effect [153]. In addition, CS also has good biodegradability, biocompatibility, and nontoxicity properties, which makes it suitable as a drug carrier. CS enjoys relatively broad research and application prospects in the field of drug carriers, and it is often used as an antitumor drug targeting carrier, sustained-release drug carrier, or gene carrier [153,154,155,156]. As a drug carrier, CS derivatives not only improve the CS solubility but also enable colon-targeted therapy of drugs and enhance drug release in alkaline environments [157]. Currently, the more commonly used CS derivative carriers include quaternized CS, thiolated CS, alkylated CS, and carboxylated CS. Common chitosan derivatives as drug carriers and their applications are shown in Table 6.

Quaternized CS has a permanent cationic charge, which can enhance the mucosal adhesion of CS and permeability of loaded drugs. For example, Wang et al. [158] demonstrated that a composite membrane composed of a quaternized CS composite hydrogel membrane, carboxymethyl cellulose composite hydrogel membrane, and 5-fluorouracil had significant toxicity against HepG2 cells, while the carboxymethyl cellulose/quaternized chitosan (CMC/HACC) composite membrane can be used as a targeted drug delivery system. In addition, Zhang et al. [159] first prepared oleoyl CS (OCH) using CS and oleoyl chloride, then prepared OCH nanoparticles by using the oil-in-water emulsification method, and finally efficiently loaded doxorubicin hydrochloride (DOX) into OCH nanoparticles. A cytotoxicity test revealed that blank OCH nanoparticles were not cytotoxic to mouse embryonic fibroblasts and human lung cancer cell line A549. Lung cancer cell proliferation experiments demonstrated that an OCH nanoparticle suspension loaded with DOX had a significantly higher inhibitory rate against A549 cells than the DOX solution. These results indicate that OCH nanoparticles are a good carrier for antitumor drugs.

In the application field of chitosan and its derivatives, its application in drug delivery has attracted the most attention of researchers, and good results have been achieved in this field. The current drug delivery system is not perfect, so it is still very important to study new drug carrier materials. Chitosan derivatives are suitable for controlled and sustained release of drugs, and researchers can focus on preparing them into microcapsules, micelles, microcapsules, and nano-drug-loading materials such as spheres.

**Table 6:** Chitosan derivatives as drug carriers and their applications.

Carrier	Drug	Application	Effect	References
Carboxymethyl-hexanoyl chitosan	Demethoxycurcumin, cisplatin	Cancer stem-like cells	Promoted synergistic effects between the drugs and were highly effective against multidrug resistance lung cancer stem-like cells	[160]
Mpeg-chitosan-oleic acid	Camptothecin	Nanomicelle	Efficiently carry hydrophobic drugs, protecting and improving their stability after oral administration	[161]
Amphiphilic chitosan (CS-DA-NAC)	Quercetin	Nanomicelle	May provide a new alternative for the effective delivery of hydrophobic drugs	[162]
Ph-sensitive *N*-naphthyl-N, *O*-succinyl chitosan	Curcumin	Colon-targeted drug delivery	Increase curcumin stability, may have potential to be a prospective candidate for curcumin delivery to the colon	[163]
Chitosan whisker grafted with oligo (lactic acid) nanoparticles	Lidocae	Transdermal drug delivery system (TDDS)	Performed as a good system for TDDS	[164]
Chitosandeoxycholic acid nanoparticles containing perfluoropentane and iron oxide	siRNA	Transdermal drug delivery system (TDDS)	Promote siRNA uptake	[165]
Methyl methacrylate modified chitosan conjugate	Curcumin	Gene and drug delivery system	An efficient target drug delivery system	[166]
*O*-Carboxymethyl chitosan	Metformin	Pancreatic cancer	Reduced colony formation ability of the cancer cells, no adverse toxicity to the organs, with anticancer potential	[167]
Carboxymethyl cellulose/quaternized chitosan composite hydrogel film	5-Fluorouracil	Hepg2 cells	Showed redox and pH responsive of drug release properties along with well biocompatibility, the drug loaded composite films with obvious toxicity against hepg2 cells	[158]
Chitosan grafted-poly (ethylene glycol) methacrylate derivative	Bevacizumab	Ophthalmic drug delivery system	A much lower dose to administer, prolonged release, the effectiveness of local delivery (which may extend up to at 14–30 days)	[168]

### 5.4. Wastewater Purification

Heavy metals, chemical reagents, dyes, and other pollutants in the water pose a serious threat to human health, which presents a great challenge for environmental maintenance efforts. At present, adsorption is the most effective and popular method for wastewater pollutant treatment [169]. CS is a natural adsorbent that can reduce turbidity, color, and small particles in water [170]. More importantly, CS can also remove heavy metal ions, microbial pollutants, and harmful pollutants (dyes, pesticides, and herbicides) in water [171,172,173]. Already authorized by the U.S. Environmental Protection Agency to be used for the purification of drinking water, CS has been used in water purification plants all over the world to remove oil, grease, heavy metals, and fine particles from water. The amino group of CS is an excellent chelating ligand with a strong attraction to metal cations, which can chelate with metal ions, such as cadmium, copper, lead, and mercury [174] and acts as the main group of CS that enables a wastewater purification effect. The application of common chitosan derivatives in wastewater purification is shown in Table 7.

At present, CS is mainly used for the removal of heavy metals in wastewater purification. The adsorption capacity of CS against heavy metals is influenced by factors such as the deacetylation degree, wastewater environment, and types of heavy metals [175]. Unfortunately, the adsorption capacity of CS is not strong enough, but CS derivatives can be synthesized via a reaction with specific substituents to improve the adsorption rate of heavy metals [176]. In addition, chemically modified CS derivatives support selective adsorption of heavy metals in wastewater. For example, Zhang et al. [177] grafted acrylic acid onto CS to synthesize new CS derivatives and then synthesized a composite adsorbent material (PAA/CTS/BC) using biochar. The study revealed that PAA/CTS/BC achieved efficient and rapid adsorption against heavy metal ions, such as Cu^2+^, Zn^2+^, Ni^2+^, Pb^2+^, Cd^2+^, Mn^2+^, Co^2+^, and Cr^3+^. The main mechanism of action is that the inner spherical complex of the carboxyl, hydroxyl, or amino group of the composite adsorptive material can complex with surface heavy metals, demonstrating strong selective absorption in the process. PAA/CTS/BC has the strongest selectivity for Cr^3+^and supports metal recovery of industrial wastewater.

In recent years, great research progress has been made in the environmental applications of chitosan and its derivative adsorbents, but their selective adsorption capacity, preparation cost, and reusability still need to be further improved.

**Table 7:** Application of chitosan derivatives in wastewater purification.

Name	Application	Effect	References
Chitosan-*O*-arginine	Removal of heavy metals	Removal of heavy metals (Mn^2+^, Pb^2+^ and Al^3+^) was 97.1%, 94.3%, and 99%	[178]
Functionalized chitosan nanoparticles with pyrimidine derivative	Removal of heavy metals	Removal of Cr (VI), Pb (II), and Cd (II) plasma from highly contaminated tannery wastewater	[179]
Carboxymethyl chitosan biochar	Removal of heavy metals	Highly selective for heavy metal ions and it also presented good stability and reusability for industrial applications	[180]
Carboxyl-rich chitosan-based flocculant (CS-g-P (AM-IA))	Flocculation and decolorization	The decolorization ability of cationic dyes by CS-g-P(AM-IA) was greatly enhanced	[181]
*N*-guanidinium chitosan/silica-containing sulphonic acid group microhybrid	Dye adsorbent	Efficient and sustainable adsorbent for methylene blue removal	[182]
Quaternary chitosan magnetite nanosorbents	The removal of glyphosate	Efficiently remove glyphosate present in realistic environmental concentrations	[183]
Chitosan-derived Schiff base	Dye adsorbent	The synthesized bio-based adsorbent material is effective for the removal of rhodamine B dye from an aqueous solution	[184]
Sulfonated chitosan (S-CS)	Dye adsorbent	The S-CS is an effective and efficient adsorbent over a wide range of pH conditions for the removal of methylene blue	[185]
Quaternized trimethyl chitosan	Water disinfection	Better antifungal effect	[186]

## 6. Conclusions and Prospects

Generally speaking, for various CS chemical modification methods, it is possible to introduce the target group into the amino group or hydroxyl group by changing the reaction conditions, but the target group is mainly introduced to the amino group. In addition, the various chemical modification methods above can basically improve the water solubility of CS. It is also possible to introduce hydrophobic groups to improve the fat solubility of CS. More importantly, CS modified by various methods achieves improved properties, especially antibacterial, antioxidant, antitumor, and hemostatic activities. Table 5 provides a comprehensive evaluation of the preparation methods and effects of the above-mentioned chemical modification technologies. Due to their excellent properties, CS and its derivatives are frequently used in wastewater treatment, textiles, wound dressings, and drug carriers, as well as in textiles, beauty and health care, cosmetics, gene therapy, sutures, bioimaging, drug carriers, and antibacterial agents. At present, there is a large number of drugs with outstanding efficacy, but their clinical applications are severely limited due to their solubility, light instability, easy oxidation, and gastrointestinal instability. In order to improve the solubility of these drugs and improve their stability, it is necessary to study and prepare more new high-quality drug carrier materials, so chemical modification of chitosan to synthesize drug carrier materials is a good direction.

Due to progress in the research on modification methods, the application range of CS and its derivatives is relatively wide. Nevertheless, the development of new applications of CS is relatively slow and has not achieved large-scale industrial application. The main reason is that CS is expensive, with a higher development price compared to petroleum-based polymers with similar properties. Therefore, in addition to strengthening development research toward improving CS modification technology, we should intensify the application research of CS derivatives, especially in the field of biomedicine. In addition, further research is needed on the grafting of CS with long-chain groups and the compounding of CS with antioxidant, antitumor, and antibacterial drugs to prepare CS derivatives with stronger activities, thus broadening its application scope. Finally, we should continually optimize the preparation of CS, investigate more economical preparation methods, and boost the large-scale production of CS.

## Figures and Tables

**Table 1:** Summary of various chemical modifications.

Modification Method	Main Reaction Site	Priority Reaction Site	Main Synthesis Method	Commonly Used Reagent	Modification Effect
Carboxylation modification	C_2_-NH_2_ C_3_-OH C_6_-OH	C_6_-OH	Direct oxidation of C_3_-OH, C_6_-OH Chloroalkanoic acid oxidation of C_2_-NH_2_	Glyoxylic acid, chloroacetic acid	Greatly improved water solubility Improved chitosan biocompatibility, antibacterial property, adsorption, etc.
Alkylation modification	C_2-_NH_2_ C_3_-OH C_6_-OH	C_2_-NH_2_	Schiff base method Phthaloyl method Reaction with halogenated alkane	Halogenated hydrocarbons, sulfate aldehydes	Improved water solubility Good biocompatibility and hemostatic properties
Acylation modification	C_2_-NH_2_ C_3_-OH C_6_-OH	C_2_-NH_2_	Acid anhydride or acid halide oxidation of -OH Acid anhydride or acid halide oxidation of C_2_-NH_2_	Acid anhydride, acid halide	Greatly improved water solubility Good biocompatibility and slow controlled drug release
Esterification modification	C_2_-NH_2_ C_3_-OH C_6_-OH	C_6_-OH	Sulfation Acylation Phosphorylation	Oxygen-containing inorganic acid, acid derivatives	Improved adsorption, antibacterial and water solubility of chitosan, etc.
Sulfonation modification	C_2_-NH_2_ C_3_-OH C_6_-OH	C_2_-NH_2_	Reaction with sulfonate	Sulfonate	Strong antibacterial activity, anticoagulant activity and good blood compatibility, etc.
Quaternary ammonium salt modification	C_2_-NH_2_ C_3_-OH C_6_-OH	C_2_-NH_2_	Reaction with halogenating reagent Reaction with quaternary ammonium salt containing alkylene oxide Grafting modification of quaternary ammonium	Methyl iodide, *N*-(3-chloro-2-hydroxypropyl) Base) trimethyl ammonium chloride	Significantly improved water solubility Excellent antibacterial activity and good antioxidant activity
Graft copolymerization modification	C_2_-NH_2_ C_3_-OH C_6_-OH	All are acceptable	Oxidative coupling copolymerization Free radical graft copolymerization Condensation copolymerization	Carbodiimide, hydrogen peroxide, ascorbic acid	Improved antibacterial, antioxidant, anti-tumor activities, etc.
Schiff base modification	C_2_-NH_2_	C_2_-NH_2_	Reaction with carbonyl compound	Fatty aldehydes, aromatic aldehydes, ketones	Significantly improved water solubility and antibacterial activity Good antioxidant, anti-tumor activities, etc.

**Table 2:** Conditions of different modification methods and their product summary.

Modification Method	Condition	Obtained Product	References
Carboxylation modification	2,4-pentadion, aminobenzoic acid, ClCH_2_COOH	*O*-carboxymethyl chitosan Schiff bases	[32]
Carboxylation modification	40 wt% NaOH, CICH_2_COOH	*O*-carboxymethyl chitosan	[33]
Carboxylation modification	chloroacetic acid, isopropanol, sodium hydroxide, methanol and hydrochloric acid	*O*-carboxymethyl chitosan	[34]
Carboxylation modification	isopropyl alcohol, NaOH, CICH_2_COOH	*N,O-*carboxymethylated chitosan	[35]
Carboxylation modification	dimethyl sulfoxide, succinic anhydride, NaOH, acetone	*N*-succinyl chitosan	[36]
Alkylation modification	*n*-butyraldehyde, NaBH_4_, trichlorodihydroxypropyl triethyl ammonium chloride	*N*-alkylated quaternary ammonium chitosan	[39]
Alkylation modification	5% 4-octadecyl benzaldehyde, ethanol, NaOH, NaBH_4_	*N*-alkylated chitosan sponge	[40]
Alkylation modification	acetic acid, ethanol, sodium cyanoborohydride, lauraldehyde	*N*-alkylated chitosan	[41]
Alkylation modification	1% acetic acid, octaldehyde, sodium borohydride, NaOH	*N*-octyl chitosan	[43]
Alkylation modification	CH_2_Cl_2_, triethylamine, acetic acid, Na (OAc)_3_BH, acetonitrile, dimethylsulfate	*N*-alkyl-*N,N-*dimethyl chitosan derivatives	[44]
Alkylation modification	chloroform, dodecanol, N_2_, *N,N*’-carbonyldiimidazole	*O*-alkylated CS	[46]
Acylation modification	trifluoroacetic acid, dichloromethane, pyridine, lauric anhydride	*O*-acylated chitosan nanofibers	[50]
Acylation modification	succinic anhydride, hydrochloric acid, and alkaline chitosan	*N*-succinyl chitosan	[51]
Acylation modification	oleic acid, arginine, sodium acetate buffer, *N*-(3-dimethylaminopropyl)-*N*-ethylcarbodiimide hydrochloride	amphiphilic chitosan	[52]
Esterification modification	dimethyl sulfoxide, folic acid, *N*-hydroxy succinimide, 1-ethyl-3-(3-dimethylaminopropyl) carbodiimide hydrochloride, N_2_	folate-modified chitosan	[58]
Esterification modification	1,4-dioxane, sulfamic acid, urea	sulfamic acid sulfation of chitosan.	[59]
Esterification modification	NaHSO_3_, NaNO_2_, *N*-succinyl chitosan, NaOH	*N*-succinyl chitosan sulfates	[60]
Esterification modification	dimethylformamide, chlorosulphonic acid, 20% NaOH	sulfated chitosan	[61]
Esterification modification	ClHSO3, *N*,*N*-dimethylformamide, formamide, formic acid, argon	2-*N*,6-*O*-sulfated CS	[89]
Esterification modification	methanesulfonic acid, phosphorus pentoxide	phosphorylated chitosan	[64]
Esterification modification	urea, *N*,*N*-Dimethylformamide, H_3_PO_4_	phosphorylated magnetic chitosan composite	[65]
Sulfonation modification	2% acetic acid, 1,3 propane sultone, 60 °C, 6 h	sulfopropyl chitosan	[69]
Sulfonation modification	water-soluble oligochitosan, Maleic anhydride, 20 wt% NaOH	sulfonated chitosan	[70]
Sulfonation modification	1% aqueous acetic acid, methanol, sulfobenzoic acid cyclic anhydride	sulfated chitosan	[71]
Quaternary ammonium salt modification	benzaldehyde, NaBH_4_, *N*-methyl-2-pyrrolidone, NaI, CH_3_I, pyridine carboxaldehyde, sodium, methyl iodide	double quaternized chitosan derivatives	[74]
Quaternary ammonium salt modification	*N*-(3-chloro-2-hydroxypropyl) trimethylammonium chloride, 50 °C, 24 h	quaternized chitosan	[75]
Quaternary ammonium salt modification	2,3-epoxypropyltrimethyl ammonium chloride, isopropanol, 80 °C, 12 h	2-hydroxypropyltrimethyl ammonium chloride chitosan	[76]
Quaternary ammonium salt modification	2,3,4,5,6-pentafluorobenzaldehyde, sodium borohydride, methanol, 2,3,4,5,6-pentafluorobenzaldehyde, glycidyl trimethylammonium chloride	fluorinated quaternary chitosan derivatives	[79]
Quaternary ammonium salt modification	benzaldehyde, QAS p-toluene sulfonate, isopropanol, 40% NaOH, 0.25 M HCl, ethanol	*O*-quaternized chitosan	[80]
Graft copolymerization modification	ferulic acid, ethanol,1-ethyl-3-(3-dimethylaminopropyl) carbodiimide hydrochloride, nitrogen gas	chitosan–ferulic acid conjugates	[82]
Graft copolymerization modification	acetic acid,1-hydroxybenzotriazole, 1-ethyl-3-(3-dimethylaminopropyl) carbodiimide hydrochloride, Gallic acid, ethanol	chitosan films grafted with gallic acid	[83]
Graft copolymerization modification	ceric ammonium nitrate, acrylonitrile, 65 °C, 4 h	polyacrylonitrile-graft-chitosan	[84]
Graft copolymerization modification	hydrogen peroxide, ascorbic acid, caffeic acid, 24 h	caffeic acid grafted chitosan	[85]
Graft copolymerization modification	2-hydroxy ethyl acrylate, potassium persulfate, N_2_	chitosan grafted 2-hydroxyethylacrylate	[86]
Schiff base reaction	4-chloro benzaldehyde, ethanol, 50 °C, 6 h	4-chloro benzaldehyde modified chitosan	[87]
Schiff base reaction	benzophenone, ethanol, 50 °C, 6 h	benzophenone-modified chitosan	[87]
Schiff base reaction	2-chloroquinoline-3-carbaldehyde, 50 °C, 10 h, NaOH	chitosan Schiff base derivatives	[88]

**Table 3:** Studies of antibacterial, antitumor, hemostatic, and antioxidant activities of different chitosan derivatives.

Activity	Name	Effect	References
Antibacterial activity	Schiff base of chitosan	Improved the antibacterial activity of chitosan, and the antibacterial activity increased with the increase of concentration	[92]
Antibacterial activity	Chitosan-sulfadiazine membrane	Improved the biodegradation rate, antibacterial ability, and healing properties of chitosan	[93]
Antibacterial activity	*O*-Quaternary ammonium *N*-acyl thiourea chitosan	Exhibited excellent solubility over a wide pH range; significantly enhanced the antibacterial activity of chitosan	[94]
Antitumor activity	Caffeic acid-conjugated chitosan	Inhibited proliferation of CT26 colon cancer cells and accelerated tumor cell apoptosis	[101]
Antitumor activity	Carboxymethyl chitosan	Significantly inhibited the growth of mouse hepatocarcinoma 22 tissues and could promote tumor cell necrosis	[102]
Antitumor activity	The sulfated benzaldehyde chitosan	Significantly inhibited the proliferation of breast cancer MCF-7 and induced apoptosis	[103]
Hemostatic activity	Composite hydroxybutyl chitosan sponge	Better water retention, and antibacterial and wound healing abilities	[109]
Hemostatic activity	*N,O-*carboxymethyl chitosans	Possessed excellent hemostasis both in vitro and in vivo	[110]
Hemostatic activity	*N*-succinyl chitosan nonwoven, carboxymethyl chitosan nonwoven, quaternized chitosan nonwoven	Exhibited a better hemostatic property than gauze and chitosan nonwoven	[111]
Antioxidant activity	*N*-quaternized and double *N*-diquaternized chitosan derivatives	The number of quaternized groups of chitosan derivatives contributes to their antioxidant activity	[114]
Antioxidant activity	Succinyl-chitosan (SC) and glutaryl-chitosan (GC)	SC and GC showed pronounced antioxidant, antiplatelet, and anticoagulant activity	[53]
Antioxidant activity	Caffeic acid-grafted chitosan, ferulic acid-grafted chitosan	Greatly enhanced the in vivo and in vitro antioxidant activity of chitosan	[116]

**Table 5:** Application of chitosan derivatives in wound therapy.

Carrier	Material	Application	Effect	References
Quaternary ammonium *N*,*N*,*N*-trimethyl chitosan derivative	Sponges	In vitro wound healing	Promoting early collagen formation and re-epithelialization in rat wounds	[141]
Thymine-modified chitosan derivatives (TC)	Sponges	In vivo wound healing	TC sponges could significantly accelerate the wound healing process compared to gauze and chitosan sponge	[142]
Carboxymethyl-chitosan	Dressings	Rat skin wound	Showing better epithelialization and healing properties in vivo	[143]
Carboxymethyl chitosan	Fabrics	Scalded rats	Accelerated angiogenesis and new collagen deposition in scalded rats	[144]
Carboxymethyl chitosan grafted with collagen	Sponges	Burn wound	Promote wound healing efficiency, enhanced cell migration, and promoted skin regeneration	[145]
Hydroxypropyl chitosan/soy protein isolate composite films	Dressings	Full-thickness skin wound in rats	May be a potential candidate as the wound dressing	[146]
Chitosan–ferulic acid-conjugated poly (vinyl alcohol) (CS–FA-PVA)	Polymer film	L929 mouse fibroblasts	72 and 100% wound closure by 25 μL of CS-FA-PVA, respectively, at 12 and 24 h	[147]
Catechol-conjugated chitosan	Tissue adhesive	Porcine tissue	Induces accelerates the wound closure and healing effects by comparison with a commercial adhesive	[148]
Hyaluronic acid/quaternized chitosan hydrogels	Hydrogels	Seawater-immersion Wound healing	Promote wound healing of seawater-immersed wounds and prevent bacterial infection	[149]
Synthesis of *N*-succinyl	Nanoparticles film	Wister rat wounds	Had excellent antimicrobial, cytotoxicity, and wound healing activity	[150]

## Data Availability

Not applicable.
